# Impact of Oxygen Stewardship Training on Knowledge and Practice for Oxygen Administration Among Healthcare Professionals Working in the Rajkot District

**DOI:** 10.7759/cureus.84152

**Published:** 2025-05-15

**Authors:** Vrinda Oza, Bhavesh Kanabar, Vandana Parmar, Kiran Piparva, Devanshi Patel, Jyoti Chauhan

**Affiliations:** 1 Anesthesiology, P.D.U. Government Medical College Rajkot, Rajkot, IND; 2 Preventive and Social Medicine, P.D.U. Government Medical College Rajkot, Rajkot, IND; 3 Pharmacology, All India Institute of Medical Science Rajkot, Rajkot, IND

**Keywords:** covid-19, hcp, hfnc, medical oxygen, oxygen stewardship

## Abstract

Aim: To evaluate the effectiveness of the oxygen stewardship training program in improving knowledge and practices regarding medical oxygen administration among healthcare professionals (HCPs) at the tertiary care teaching hospital.

Methods: A cross-sectional study was conducted among HCPs who participated in the oxygen stewardship training program at P.D.U. Government Medical College, Rajkot, India, in 2022. Pretest and posttest data were collected using a questionnaire to evaluate changes in their knowledge and practices regarding oxygen administration and analyzed with appropriate tests.

Results: A total of 50 HCPs participated in the study, with the majority aged 31-40 years (86%) and 56% being nurses. Before training, knowledge on oxygen stewardship, oxygen therapy criteria, device selection, and technical systems varied, with only 18 (47.4%) aware of initiation criteria and 13 (26%) aware of facility-wise oxygen demand estimation. Awareness of pressure swing adsorption plant (PSA), Petroleum and Explosives Safety Organization (PESO), and Pin Index Safety System (PISS) was moderate to low. Post-training, significant improvements were observed: knowledge of oxygen therapy criteria rose to 36 (70.3%, p=0.044), oxygen device selection to 45 (90%, p=0.014), and demand estimation to 31 (61.2%, p<0.001). Awareness of PSA, PESO, and PISS also increased. Practice-related improvements were notable: oxygen prescription practices (82%-100%, p=0.002), correct mask fitting (88%-100%, p=0.001), high-flow nasal cannula (HFNC) use (64%-96%, p<0.001), and ventilator handling (64%-100%, p<0.001). These findings suggest the training program effectively enhanced both knowledge and practical application.

Conclusion: The oxygen stewardship training significantly improved HCPs' knowledge and practices, though additional focus on PSA systems, demand estimation, and ventilator management is needed to sustain these gains.

## Introduction

The COVID-19 pandemic exposed critical weaknesses in healthcare systems worldwide, with medical oxygen becoming a vital yet limited resource, especially in low- and middle-income countries (LMICs), where over half a million COVID-19 patients required daily oxygen treatment [1]. Medical oxygen, essential for treating various health conditions and recognized by the World Health Organization (WHO) as a vital medicine, had previously received little attention [2]. The COVID-19 pandemic has highlighted and accelerated existing inequities in oxygen supply and delivery [3]. The unprecedented demand for medical oxygen during the pandemic's peak, particularly during India's devastating second wave in 2021, highlighted the urgent need for efficient oxygen management and capacity building among healthcare workers [4,5]. This crisis acted as a catalyst for the development and implementation of comprehensive oxygen stewardship programs, marking a paradigm shift in how medical oxygen is managed in healthcare settings [4,5].

The Government of India initiated the National Oxygen Stewardship Programme in December 2021, representing one of the first systematic attempts globally to standardize oxygen management practices [6]. The concept of "oxygen stewardship” emerged as a structured approach to optimize oxygen utilization, encompassing several critical components: proper prescription practices, selection of appropriate delivery devices, monitoring protocols, prevention of wastage, and maintenance of safety standards and sustainable supply chains to ensure patient safety [6,7]. Each of these elements requires specific knowledge and skills, making comprehensive training essential for healthcare workers involved in oxygen therapy management [8]. This program aimed to create a cadre of trained healthcare professionals capable of managing oxygen resources effectively while ensuring optimal patient care, particularly in resource-limited settings [3]. The WHO emphasizes that effective oxygen therapy involves not just the availability of oxygen but also the correct assessment of need, appropriate delivery methods, and regular monitoring of patient response [9]. Previous studies have demonstrated significant variations in healthcare workers' knowledge and practices regarding oxygen therapy [10]. Research from various settings has shown that inadequate understanding of oxygen delivery systems, monitoring requirements, and safety protocols can lead to suboptimal patient care and resource wastage [11]. Furthermore, studies have indicated that structured training programs can significantly improve healthcare workers' competency in oxygen therapy management. The current study was planned to impact of “oxygen stewardship” training on knowledge and practice for oxygen administration of health care professionals working at the Rajkot district level.

## Materials and methods

Study design: Retrospective observational study.

Study setting: Data were obtained from the records of the Oxygen stewardship training program conducted at P.D.U. Government Medical College, Rajkot, India, in 2022.

Ethical consideration: The study was started after institutional ethical clearance.

Study participants: Selected healthcare professionals (nursing staff, medical officers, class 1 officers) who were engaged in oxygen administration and working in a government setup.

Study process: The two-day Oxygen Stewardship Training Program comprised a total of seven lecture-based sessions delivered by subject experts, addressing six key topic areas essential for comprehensive oxygen management. The training employed a diverse range of participatory learning approaches to enhance engagement and knowledge retention. These methodologies included interactive presentations, case studies, two group discussions, a dedicated hands-on session, and the use of visual aids such as flowcharts, oxygen equipment, and safety signage.

The core content areas covered included the following: (1) an introduction to oxygen therapy and its critical role in respiratory care management; (2) oxygen delivery solutions, including production sources, storage systems, and monitoring equipment; (3) oxygen inventory management and demand calculation techniques; (4) rational use of oxygen, supported by oxygen audit processes and related tools; (5) medical gas handling along with essential safety protocols; and (6) an overview of the respiratory care market and the supplier landscape.

In addition to the seven lectures, two group discussion segments were embedded within key sessions-particularly those focused on the rational use of oxygen and oxygen audits - encouraging participants to share experiences and best practices from their respective healthcare settings. A dedicated hands-on interactive session was conducted, allowing participants to engage directly with oxygen therapy devices and observe the functioning of key infrastructure, including liquid medical oxygen, medical gas handling, and pressure swing adsorption plants.

The training also included pre- and post-training assessments to evaluate knowledge gain and gather participant feedback, forming the basis for measuring the program's impact within the study’s methodology. By combining practical, interactive teaching methods with comprehensive content coverage, the training program aimed to empower participants to implement effective oxygen management strategies in their healthcare settings-ultimately contributing to improved patient care and optimized resource utilization. The training was associated with both pre- and post-training assessments to evaluate knowledge gain and gather participant feedback.

Data collection tool: A questionnaire for assessment of their knowledge and practice towards oxygen administration was prepared from the scientific literature and training modules [3,7,12]. The questionnaire contains open and closed-ended questions and was pretested and validated by experts. The questionnaire was distributed, and filled-in forms were collected immediately before the start of the training session (pretest). On day second day, at the end of the training, the same form was circulated and the filled-up forms were collected immediately (posttest).

Data analysis plan: Sociodemographic data and data related to participants' knowledge and practice of oxygen administration were entered into Microsoft Excel and analyzed using descriptive statistics (frequency and percentage). Comparisons between pre-test and post-test data on knowledge and practice were conducted using the chi-square test using statistical software Jamovi (version 2.4.8; https://www.jamovi.org/).

## Results

A total of 50 healthcare professionals (HCPs) who were involved in oxygen administration and worked within the government healthcare setup, and for whom complete data were available, were included in the study. Forty-three (86%) participants were aged 31-40 years, 30 (60%) were male, and 28 (56%) were nurses, followed by 10 (20%) medical officers and eight (16%) anesthesiologists by profession (Table 1).

**Table 1:** Demographic profile of the study participants (N=50)

Demographic profile		No.	%
Age groups (years)	≤30	20	40
	31-40	23	46
	41-50	05	10
	51-60	02	04
Gender	Male	30	60
	Female	20	40
Profession	Nursing	28	56
	Medical officers (MBBS)	10	20
	Anaesthesiologist (MD)	8	16
	Another speciality (MD)	4	12
No. of Experience (years)	<5	10	20
	5-10	26	52
	>10	14	28

Before training, awareness of the Oxygen Stewardship Programme was observed in 40 (80%) study participants. Knowledge about medical oxygen administration - namely, initiation criteria, selection of appropriate devices based on patient needs, use of oxygen humidifiers, and side effects of medical oxygen - was observed in 18 (47.4%), 34 (68%), 45 (90%), and 46 (92%) participants, respectively. Technical knowledge regarding oxygen storage, types of oxygen cylinders, and facility-wise oxygen demand estimation was observed in two (5.3%), 36 (72%), and 13 (26%), respectively.

Awareness of pressure swing adsorption (PSA), Petroleum and Explosives Safety Organization (PESO), and Pin Index Safety System (PISS) was observed in 36 (72%), 44 (88%), 25 (50%), and 25 (50%) participants, respectively, before the training. Forty-six (92%) study participants were aware of the audit of the oxygen administration system before training.

Following the training program, knowledge related to the National Oxygen Stewardship Programme improved significantly, increasing from 28 (55.1%) to 39 (76.6%) (p=0.027). Understanding of COVID-19 oxygen therapy criteria also showed a marked improvement, rising from 18 (47.4%) to 36 (70.3%) (p=0.044), while understanding of oxygen device selection criteria rose from 34 (68%) to 45 (90%) participants (p=0.014). Knowledge regarding the operation of the highest oxygen delivery devices increased from 44 (88%) to 50 (100%) (p=0.035). Technical knowledge related to facility-wise oxygen demand estimation significantly improved from 13 (26%) to 31 (61.2%) participants (p<0.001). Post-training knowledge of key oxygen systems also showed improvement: awareness of PSA increased from 44 (88%) to 49 (98%) participants (p=0.083), PESO from 25 (50%) to 38 (76%) participants (p=0.007), and PISS from 25 (50%) to 33 (66%) participants (p=0.091).

However, the assessment also identified knowledge gaps requiring additional attention, particularly in technical areas such as non-rebreathing bag mask (NRBM) functionality (34% correct post-training), about the use of an oxygen humidifier (18% correct post-training) (Table 2).

**Table 2:** Comparison of pretest and posttest responses of HCPs on the knowledge-related questions (N=50) χ²=chi-square statistic, df=degree of freedom, HCPs=healthcare professionals

Sr. No.	Questions: Knowledge about	Response (correct/incorrect)	Pretest response (%)	Posttest response (%)	Total	Statistical test (p-value)
1	What is the “National Oxygen Stewardship Programme”?	Correct	40 (80.0)	41 (82.0)	81	χ²=0.065, df=1, p=0.799
		Incorrect	10 (20.0)	9 (18.0)	19	
2	Do you know the criteria for initiation of oxygen therapy in COVID-19 patients?	Correct	18 (47.4)	26 (70.3)	44	χ²=4.055, df=1, p=0.044
		Incorrect	20 (52.6)	11 (29.7)	31	
3	What are the criteria for the selection of an oxygen device in patient care?	Correct	34 (68.0)	45 (90.0)	79	χ²=6.028, df=1, p=0.014
		Incorrect	16 (32.0)	5 (10.0)	21	
4	Do you know about the use of oxygen humidifiers?	Correct	4 (8.0)	9 (18.0)	13	χ²=2.210, df=1, p=0.137
		Incorrect	46 (92.0)	41 (82.0)	87	
5	What are the parameters required to assess adequate oxygenation in a patient?	Correct	45 (90.0)	48 (96.0)	93	χ²=0.814, df=1, p=0.433
		Incorrect	5 (10.0)	2 (4.0)	7	
6	What are the side effects of medical oxygen therapy?	Correct	46 (92.0)	40 (80.0)	86	χ²=2.076, df=1, p=0.150
		Incorrect	4 (8.0)	10 (20.0)	14	
7	Do you know about the types of oxygen cylinders?	Correct	36 (72.0)	41 (82.0)	77	χ²=1.412, df=1, p=0.235
		Incorrect	14 (28.0)	9 (18.0)	23	
8	What is a “Pressure Swing Adsorption plant (PSA)”?	Correct	44 (88.0)	46 (92.0)	90	χ²=0.444, df=1, p=0.505
		Incorrect	6 (12.0)	4 (8.0)	10	
9	What is the “Petroleum and Explosives Safety Organization (PESO)”?	Correct	25 (50.0)	38 (76.0)	63	χ²=7.250, df=1, p=0.007
		Incorrect	25 (50.0)	12 (24.0)	37	
10	What is “Pin Index Safety System (PISS)”?	Correct	25 (50.0)	35 (70.0)	60	χ²=3.375, df=1, p=0.066
		Incorrect	25 (50.0)	15 (30.0)	40	
11	Do you know about “dura oxygen (liquid medical oxygen) storage?	Correct	2 (5.3)	0 (0.0)	2	χ²=0.487, df=1, p=0.485
		Incorrect	36 (94.7)	37 (100.0)	73	
12	Do you know about the elements of the oxygen audit?	Correct	46 (92.0)	46 (92.0)	92	χ²=0.000, df=1, p=1.000
		Incorrect	4 (8.0)	4 (8.0)	8	
13	Application used in estimating facility-wise oxygen demand	Correct	13 (26.0)	30 (61.2)	43	χ²=12.498, df=1, p=0.000
		Incorrect	37 (74.0)	19 (38.8)	56	

Only 10 (20%) study participants had received prior training regarding oxygen administration. A total of 41 (82%) participants reported that the practice of writing prescriptions for medical oxygen with mention of flow rate and endpoint was followed in their healthcare setup. The majority (44, 88%) indicated that they ensured appropriate mask size selection and optimum fitting while the patient was on an NRBM. Additionally, 32 (64%) participants reported guiding patients to keep their mouths closed while on a high-flow nasal cannula (HFNC).

The training led to significant improvements in practices, including checking oxygen prescriptions (41 (82%) to 50 (100%), p=0.002), correct mask size usage (44 (88%) to 50 (100%), p=0.001), HFNC utilization (32 (64%) to 48 (96%), p<0.001), ventilator management (32 (64%) to 50 (100%), p<0.001), valve closure in pipeline systems (41 (82%) to 50 (100%), p=0.002), and regular leak checks (38 (76%) to 49 (98%), p=0.001) (Table 3).

**Table 3:** Comparison of pretest and posttest responses of HCPs on the practice-related questions (N=50) *Criteria for incorrect responses=SPO_2_ > 92 for > 12/24 hours OR SpO_2_ > 94 for > 24 hours; HCPs=healthcare professionals

Sr. No.	Questions	Response (correct/incorrect)	Pretest response (%)	Posttest response (%)	Total	Statistical test (p-value)
1	Have you received training for medical oxygen administration before?	Yes	10 (20)	50 (100)	60	χ²=66.7, df=1, p<0.001
		No	40 (80)	0	40	
2	Is the practice of writing oxygen prescription with mention of flow rate and end point being followed at your hospital?	Yes	41 (82)	50 (100)	91	χ²=9.89 (100), df=1, p=0.002
		No	9 (18)	0	09	
3	When do you start the escalation of oxygen administration? Correct response (SpO2>94 for more than 12 hrs) & rest were incorrect response*	Yes	12 (24)	40 (80)	52	χ²=31.4, df=1, p<0.01
		No	38 (76)	10 (20)	48	
4	Do you ensure the usage of the right size of mask?	Yes	44 (88)	50 (100)	94	χ²= 6.38, df=1, p=0.001
		No	6 (12)	0	6	
5	Do you encourage the patient to keep their mouth closed when he/she is on HFNC?	Yes	31 (62)	48 (96)	79	χ²=17.4, df=1, p<0.001
		No	19 (38)	2 (40)	21	
6	Do you check the optimum fitting of the mask while using the non-rebreathing bag mask (NRBM)?	Yes	44 (88)	50 (100)	94	χ²=6.38, df=1, p=0.012
		No	6 (12)	0	6	
7	Do you ensure closure of valves in pipeline system in "No use areas”?	Yes	41 (82)	50 (100)	91	χ²=9.89, df=1, p=0.002
		No	9 (18)	0	9	
8	Do you switch the ventilator to standby mode during the feeding of patient?	Yes	32 (64)	50 (100)	82	χ²=22, df=1, p<0.001
		No	18 (36)	0	18	
9	Do you check appropriateness of flow rate as per oxygen therapy modality (NP/Face mask/NRBM/Bipap)?	Yes	35 (70)	50 (100)	85	χ²=17.6, df=1, p<0.001
		No	15 (30)	0	15	
10	Do you check oxygen leaks regularly?	Yes	38 (76)	49 (98)	87	χ²=10.7, df=1, p=0.001
		No	12 (24)	1 (2)	13	

## Discussion

The COVID-19 pandemic in 2021 significantly accelerated the global demand for oxygen supplies. To effectively manage the increased demand for oxygen, the stewardship program prioritized targeted training for healthcare professionals, aiming to enhance their knowledge and skills for oxygen administration to ensure its rational use and minimize wastage [5,6].

It was observed that younger participants, particularly nursing staff, participated in training as they strongly recognized the professional need to learn and adapt to the training. A similar finding was also reported in a multicentric study conducted by Demilew et al. [13]. The diverse professional background of participants, including specialists, medical officers, and nursing staff, provided a comprehensive assessment platform, similar to other successful medical training interventions reported in the literature [13,14]. According to a meta-analysis conducted by Aynalem et al., a significant correlation was found between knowledge of oxygen therapy and its practical application, suggesting that healthcare professionals with a strong understanding of oxygen therapy are more likely to perform effectively in practice [15].

The significant enhancement in the knowledge and practical management of patients on a ventilator (64%-100%) was observed in the post-training session. These improvements suggest that the program's hands-on, practical approach effectively addressed critical safety protocols, aligning with best practices recommended in international guidelines. The program's success in improving practical skills more substantially than theoretical knowledge reflects findings from other healthcare training initiatives [10,16].

The findings have several important implications for healthcare systems, particularly in resource-constrained settings. First, the successful improvement in oxygen demand estimation and management skills addresses a critical gap identified during the COVID-19 pandemic. Second, the standardization of practical protocols achieved through the training could contribute to more efficient resource utilization and improved patient safety.

Study limitation

Several limitations should be considered when interpreting these results. The single-center nature of the study and the relatively small sample size (N=50) may limit generalizability. The immediate post-training assessment, while showing promising results, does not provide information about the long-term retention of knowledge and skills, a limitation noted in similar healthcare training studies.

Recommendation

To enhance healthcare training, it is essential to implement regular refresher courses to reinforce and update knowledge, especially in technical areas. Additionally, more intensive modules should be developed for complex technical concepts, alongside the integration of simulation-based training for high-risk procedures. Continuous monitoring mechanisms should be established to ensure sustained improvements in practice. Moreover, creating specialized training modules tailored to the needs of different healthcare professional categories will ensure that the training is relevant and effective for all levels of staff.

Future research needs

Future studies should explore ways to enhance the understanding and effectiveness of oxygen stewardship training. Research should assess the long-term retention of knowledge and skills acquired through the training program. Additionally, a cost-effectiveness analysis is needed to determine the financial viability and value of implementing such training on a larger scale.

Comparative studies involving different training methodologies could help identify the most efficient and engaging approaches. Finally, future research should also examine the scalability of the program across various healthcare settings to determine its broader applicability and sustainability.

## Conclusions

The program demonstrated substantial success in enhancing participants' knowledge of oxygen administration in patient care, as well as its management in the hospital setting. By evaluating changes in participants' understanding and skills through pre- and post-training assessments, this research offers valuable insights into the program's effectiveness and highlights areas that may require further attention. These findings are useful for refining future oxygen stewardship training initiatives and suggest that implementing regular refresher courses could help sustain and further improve the knowledge and practices gained during the training.

## Appendices

Impact of “Oxygen Stewardship Training” On Knowledge and Practice for Oxygen Administration among Health Care Professionals Working in the Rajkot District

                                                                                         Questionnaire

Age: _____                                                                                               

Gender:  Male/Female                

Education: MBBS/MD/Nursing _______________

Current job_______________________                         Experience (No. of years) __

Encircle the correct answers of the following questions ( The correct answers are indicated in bold)

1.  Do you know about the “National Oxygen Stewardship Program” in India?   YES /NO

2.    Have you taken training for the administration of oxygen before?   YES/N

  3.  Which of the following are the criteria for Oxygen device selection?

          a. **Delivery system compatibility**

          b. Quality: Certifications and Registration.

          c. Functional Requirements: Critical elements for use.

          d. Competitive pricing, prepayment, and potential procurement

4.  Use of oxygen humidifiers are necessary when__________

           **a**. **When oxygen is delivered at higher-than-standard flow rates**

           b. When methods of oxygen delivery bypass the nose

           c. When oxygen is delivered at standard flow rates

5. Do you know about the types of oxygen cylinders?    YES/NO

6.  What is PSA?

          ** a**. **Power Swing Adsorption plant**

           b. Pressure Swing Adsorption plant

           c. Pressure Swing automatic plant

7. What is the full form of PESO?

           a. Petrol and Explosives Safety Organization 

           **b.** **Petroleum and Explosives Safety Organization**

           c. Preventive and Essential Services Organization

           d. Petroleum and Expensive Society on Oxygen

8.  What is Pin Index Safety System (PISS)?

           a. Correct cylinder size for medical gas

           b. Built-in integral pressure regulator

          ** c.** **The correct gas is connected to the correct regulator and equipment**

           d. Indicate the valve size of the regulator

9.  What are the criteria for starting oxygen therapy in moderate to severe COVID-19 patients, and how many litres of oxygen per minute should be started initially?

           **a.** **SpO2 < 90% and 5Liter/minute **      

           b. SpO2 < 94-92 % and 5Liter/minu     

           c. SpO2 < 92% and 5Liter/minute         

           d. SpO2 < 89% and 5Liter/minute

10.  Which of the following oxygen devices delivers the highest oxygen (L/min)?

           a. Simple face mask          

           **b.** **NRBM   **                           

           c. Nasal prong

11.  Nasal Reservoir Bag Mask (NRBM) is a high-flow oxygen-delivering device.

           a. True

           b. False

12.  Do you check the optimum fitting of the mask while using a non-rebreathing bag mask (NRBM)? YES/NO

13.  Which parameters are required to check in patients for adequate oxygenation?

          a. SpO2                    

          b. ABG (arterial blood gas analysis)                         

          c.  **Both**

14.  Do you ensure the closure of valves in the pipeline system in "No use areas"?  YES/NO

15.  Do you switch the ventilator to standby mode during the feeding of a patient? YES /NO

16.  Do you encourage the patient to keep their mouth closed when on HFNC?    YES /NO

17.  Which of the following application is used in estimating facility wise                   oxygen demand and escalating the demand to the authorities?

          a. OxyCare Management Information System           

          b. Oxygen Digital Tracking System

          c. **Oxygen Demand Aggregation System    **               

          d. Oxygen Management Tracker

18.  Dura oxygen (liquid medical oxygen) storage should be ________

         a. Should be covered with a top                                     

         b. Should not be exposed to sunlight

         c. Should be exposed to sunlight                                 

         d. **(a+b)**

19.  Which of the following are elements of the oxygen audit?

         a. Records                                                                      

         b. Physical verification

         c. Documentation                                                           

         d. **All of the above**

20.  Who should do an audit for oxygen management at the hospital?

         a.  District collector representative                                

         b. FDA supervisor

         c.  Anesthetist                                                                  

         **d. Any of the above**

21.  Is the practice of writing oxygen prescriptions with mention of flow rate and endpoint being followed at your hospital?   YES/NO

22.  Which of the following is a side effect of medical oxygen therapy?

          a. Chest pain                                                                   

          b. Nasal bleeding

          c. Reperfusion injury                                                      

          **d. All of the above**
